# Supplementary material for: Empirical methods for controlling false positives and estimating confidence in ChIP-Seq peaks
Source: BMC Bioinformatics. 2008 Dec 5;9:523. doi: 10.1186/1471-2105-9-523 (PMC2628906; doi:10.1186/1471-2105-9-523)
Supplement: Additional File 1 — A variety of html documents from the USeq web site detailing the available applications, their best usage, output file type descriptions, command line menus, etc. [file 1471-2105-9-523-S1.zip › USeqUserGuides/JavaDoc.zip!/JavaDoc/index-files/index-1.html]

A-Index


|  |  |  |
| --- | --- | --- |
| **Overview** \| Package \| Class \| Use \| **Tree** \| **Deprecated** \| **Index** \| **Help** | |  |
| PREV LETTER   **NEXT LETTER** | **FRAMES**    **NO FRAMES**     **All Classes** |  |


A B C D E F G H I J L M N O P Q R S T U V W X Z 

---


## **A**

**AccountManagement** - Class in bioroot: **AccountManagement()** - Constructor for class bioroot.AccountManagement: **actionPerformed(ActionEvent)** - Method in class gata.aligner.BrowseButtonAction: **actionPerformed(ActionEvent)** - Method in class gata.menu.SaveImagePanel: **actionPerformed(ActionEvent)** - Method in class util.apps.gump.GumpToolPanel: **add(int, float)** - Method in class edu.utah.seq.data.HeatMapMakerPosNeg: Adds a line to the global ArrayLists. **add(int, float)** - Method in class trans.main.WindowBlockMakerTwoColor: Adds a gr line to the global ArrayLists for later burning to bar. **addAction(JButton, int)** - Method in class gata.plotter.ToolsFrame: **addAnAntibodyPrep(AntibodyBean, DBUtil)** - Static method in class bioroot.antibody.NewAntibody: Adds a new antibodyPrep to the AntibodyBean **addBlankLines(AntibodyBean, String[], int)** - Static method in class bioroot.antibody.AntibodySpreadSheet: Adds "" to items base on the number of preps and uses in the antibody bean. **addError(String)** - Method in class edu.utah.seq.data.Simulator: Make sure your read length doesn't exceed the length of the error **addExpectedSubSeq(int)** - Method in class selex.SelexParams: **addFlashCard(Flash.FlashCard)** - Method in class util.apps.Flash: Adds flash card to the end of the array. **addHeatMapBlocks(int, int)** - Method in class edu.utah.seq.data.HeatMapMakerPosNeg: Make a heatmap blocks from given window indexes, stop not included. **addHeatMapBlocks(int, int)** - Method in class trans.main.WindowBlockMakerTwoColor: Make a heatmap blocks from given window indexes, stop not included. **addIt()** - Method in class edu.utah.seq.analysis.EnrichedRegionMaker: **addIt()** - Method in class trans.main.IntervalMaker: **addIt()** - Method in class trans.main.MultiWindowIntervalMaker: **addIt()** - Method in class trans.main.SetNumberIntervalMaker: **addLabel(ArrayList)** - Method in class gata.main.AnnoSpecParams: **addNormalDNALines(ArrayList)** - Method in class gata.main.AnnoSpecParams: **addObservedSubSeq(int)** - Method in class selex.SelexParams: **addObsExpRatio(double)** - Method in class selex.SelexParams: **addOldUserAttributes()** - Method in class bioroot.UserRegistrationBean: **addPairsToCorrelate(float[], float[])** - Method in class util.gen.PearsonCorrelation: Use this method to sequentially add pairs for correlation. **addParent(StrainBean, HashMap, DBUtil, UserBean)** - Static method in class bioroot.strain.NewStrain: **addPermutatedScoresToHistogram(ArrayList)** - Method in class trans.main.ScanChip: Uses random permutation of the cel file labels to estimate a null distribution, store random scores in a histogram for p-value and FDR estimations. **addPermutatedScoresToHistogram(ArrayList)** - Method in class trans.main.ScanChromosomes: Uses random permutation of the cel file labels to estimate a null distribution, store random scores in a histogram for p-value and FDR estimations. **addPermutatedScoresToHistogram(ArrayList)** - Method in class trans.main.ScanChromosomesCNV: Uses random permutation of the cel file labels to estimate a null distribution, store random scores in a histogram for p-value and FDR estimations. **addPlasmid(AntibodyBean, HashMap, DBUtil, UserBean)** - Static method in class bioroot.antibody.AntibodyBase: **addPlasmid(StrainBean, HashMap, DBUtil, UserBean)** - Static method in class bioroot.strain.NewStrain: **addProteinLines(ArrayList, ArrayList)** - Method in class gata.main.AnnoSpecParams: **addRNALines(Line2D.Double, ArrayList)** - Method in class gata.main.AnnoSpecParams: **addScaleRuler(JMenu, String)** - Method in class gata.menu.GATAFrame: **addScaleTrackColors(JMenu)** - Method in class gata.menu.GATAFrame: **addShowHide(JMenu, String, boolean)** - Method in class gata.menu.GATAFrame: **addShowHideLabel(JMenu, String)** - Method in class gata.menu.GATAFrame: **addShowHideWithPath(JMenu, String, String)** - Method in class gata.menu.GATAFrame: **addStrain(AntibodyBean, HashMap, DBUtil, UserBean)** - Static method in class bioroot.antibody.AntibodyBase: **addStrain(PlasmidBean, HashMap, int, DBUtil)** - Static method in class bioroot.plasmid.NewPlasmid: **addThinDNALines(ArrayList)** - Method in class gata.main.AnnoSpecParams: **addTrack(JMenu, String, boolean)** - Method in class gata.menu.GATAFrame: **addUseDilution(AntibodyBean, DBUtil, int)** - Static method in class bioroot.antibody.NewAntibody: Adds a new UseDilution to the indicated AntibodyPrep **addUseDilutionBlankLines(boolean, String[], AntibodyPrep, int, DBUtil)** - Static method in class bioroot.antibody.AntibodySpreadSheet: Adds "" to items based on the number of use dilutions present in the Antibody Prep bean. **addZeros(String)** - Static method in class util.gen.Misc: Prepends zeros to a string such that its total length = 10. **addZeros(String[])** - Static method in class util.gen.Misc: Prepends zeros to each String such that its total length = 10. **ADFDataBaseSplitter** - Class in util.bio.parsers: **ADFDataBaseSplitter()** - Constructor for class util.bio.parsers.ADFDataBaseSplitter: **AggregatePlots** - Class in trans.graphics: For each region finds the underlying scores from the chromosome specific bar files. **AggregatePlots(String[])** - Constructor for class trans.graphics.AggregatePlots: **align(char[], char[])** - Method in class util.bio.parsers.BlastCompompositeGenerator: **AlignedBaseCounter** - Class in util.bio.converters: Counts and parses the bases in a multiple alignment where each string is broken into three sections like gatcc GGCCAATT cgatcca, ccat GGCCAATT cctttt, like the output from meme the central portion is aligned, the left and right flanks are not. **AlignedBaseCounter()** - Constructor for class util.bio.converters.AlignedBaseCounter: **AlignerInputForm** - Class in gata.aligner: **AlignerInputForm(GATAligner)** - Constructor for class gata.aligner.AlignerInputForm: **AlignerInputPanel** - Class in gata.aligner: **AlignerInputPanel(JFrame, GATAligner)** - Constructor for class gata.aligner.AlignerInputPanel: Complex class containing most everything to show and process the GATAligner inputs. **AlignerPreferences** - Class in gata.aligner: **AlignerPreferences()** - Constructor for class gata.aligner.AlignerPreferences: **AlignerThread** - Class in gata.aligner: **AlignerThread(ProgressMonitor, AlignerInputForm)** - Constructor for class gata.aligner.AlignerThread: **Alignment** - Class in gata.aligner: **Alignment(String, int, int, String, int, int, int, int, AlignParams, LocalAlignment)** - Constructor for class gata.aligner.Alignment: **Alignment** - Class in util.bio.calc: Methods associated with alignments, pvalues, blast. **Alignment()** - Constructor for class util.bio.calc.Alignment: **AlignPanel** - Class in gata.plotter: **AlignPanel(GATAParams)** - Constructor for class gata.plotter.AlignPanel: **AlignParams** - Class in gata.aligner: **AlignParams()** - Constructor for class gata.aligner.AlignParams: **AlignParams(AlignerPreferences)** - Constructor for class gata.aligner.AlignParams: **all4BaseCombinations** - Static variable in class util.bio.seq.Seq: All possible 4 base combinations. **ALToLine2DArray(ArrayList)** - Static method in class gata.main.GATAUtil: **ALToString(ArrayList)** - Static method in class gata.main.GATAUtil: **ALToString(ArrayList)** - Static method in class util.bio.annotation.GeneGroup: **Amplicon** - Class in edu.utah.seq.methylation454: **Amplicon(String)** - Constructor for class edu.utah.seq.methylation454.Amplicon: **AnalysisBean** - Class in cgar.beans: **AnalysisBean()** - Constructor for class cgar.beans.AnalysisBean: **AnalysisGroupBean** - Class in cgar.beans: **AnalysisGroupBean()** - Constructor for class cgar.beans.AnalysisGroupBean: **AnalysisProtocolBean** - Class in cgar.beans: describes how the analysis was conducted, provide means to upload a flat file, use Analysis.protocolNotes to describe any deviations or particulars from uploaded file for a particular experiment. **AnalysisProtocolBean()** - Constructor for class cgar.beans.AnalysisProtocolBean: **AnalysisTypeBean** - Class in cgar.beans: **AnalysisTypeBean()** - Constructor for class cgar.beans.AnalysisTypeBean: **AnnoSpecParams** - Class in gata.main: **AnnoSpecParams()** - Constructor for class gata.main.AnnoSpecParams: **AnnotateRegions** - Class in trans.anno: For annotating Intervals or binding regions with gene information, proximity, neighbors, and bias based on random trials. **AnnotateRegions(String[])** - Constructor for class trans.anno.AnnotateRegions: **AnnotateRegionsWithGeneList** - Class in trans.anno: For annotating a picks file (chr, start, stop) with gene information for a particular list of CG names. **AnnotateRegionsWithGeneList(String[])** - Constructor for class trans.anno.AnnotateRegionsWithGeneList: **Annotation** - Class in gata.geneGlyphs: **Annotation(AnnoSpecParams, GATAParams)** - Constructor for class gata.geneGlyphs.Annotation: **AntibodyBase** - Class in bioroot.antibody: **AntibodyBase()** - Constructor for class bioroot.antibody.AntibodyBase: **AntibodyBean** - Class in bioroot.antibody: **AntibodyBean()** - Constructor for class bioroot.antibody.AntibodyBean: **AntibodyBean(ResultSet)** - Constructor for class bioroot.antibody.AntibodyBean: **AntibodyBean(int, DBUtil)** - Constructor for class bioroot.antibody.AntibodyBean: Assumes id is not zero **AntibodyModify** - Class in bioroot.antibody: Servlet to modify antibodys using antibodyBeans referenced from a Session object. **AntibodyModify()** - Constructor for class bioroot.antibody.AntibodyModify: **AntibodyPrep** - Class in bioroot.antibody: **AntibodyPrep(int, ResultSet)** - Constructor for class bioroot.antibody.AntibodyPrep: **AntibodyPrep()** - Constructor for class bioroot.antibody.AntibodyPrep: **antibodyPrepFeatureNames** - Static variable in class bioroot.antibody.AntibodyBase: Used to see if any antibody preps are called for spreadsheet. **AntibodySpreadSheet** - Class in bioroot.antibody: **AntibodySpreadSheet()** - Constructor for class bioroot.antibody.AntibodySpreadSheet: **AntibodyViewPreferences** - Class in bioroot.antibody: **AntibodyViewPreferences()** - Constructor for class bioroot.antibody.AntibodyViewPreferences: **antiLog(double, double)** - Static method in class util.gen.Num: Converts to unlogged value. **antiLog(float[], int)** - Static method in class util.gen.Num: Converts an array float[] to unlogged values. **anyNaNs(IntensityFeature[])** - Static method in class trans.cel.CelProcessor: Looks for NaN in IntensityFeatures. **anyNaNs(float[])** - Static method in class trans.cel.CelProcessor: Looks for NaN in IntensityFeatures. **ap** - Variable in class gata.aligner.Blast: **append(String)** - Method in class util.gen.TextFrame: **appendDouble(double[], double)** - Static method in class util.gen.Num: Returns a new array with the newValue appended on the end. **appendLength(int)** - Method in class selex.SelexParams: **appendMemeParserReport(String)** - Method in class meme.MemeResults: **appendMemeReports(String)** - Method in class meme.MemeResults: **appendMessages(String)** - Method in class bioroot.ReagentBean: **appendMotifSearchResults(String)** - Method in class meme.MemeResults: **appendMummerHits()** - Method in class trans.tpmap.MummerMapper: Runs through mummer results and appends any hits to filtered1lq **appendNotes(String)** - Method in class trans.qc.CelFileStats: **appendPlasmidBeans(HttpSession, PlasmidBean[])** - Static method in class bioroot.plasmid.PlasmidUpload: Sets plasmidBeans attribute by appending any new beans to the beginning. **appendSelexOligoFiles(String)** - Method in class meme.MemeResults: **appendStrainBeans(HttpSession, StrainBean[])** - Static method in class bioroot.strain.StrainUpload: Sets strainBeans attribute by appending any new beans to the beginning. **appendSub(String)** - Method in class selex.SelexParams: **appendText(String)** - Method in class util.apps.gump.GumpResultsScrollPane: **appendToEight(boolean[])** - Static method in class util.bio.seq.BinaryBooleanReaderWriter: Copies and increases the size of the boolean[] to be divisible by 8. **Archive** - Class in bioroot: **Archive()** - Constructor for class bioroot.Archive: **areGenericLabelsVis()** - Method in class gata.main.GATAParams: **areLabelsVis()** - Method in class gata.main.GATAParams: **arrayList2IntArrayArray(ArrayList)** - Static method in class util.gen.Num: Converts an ArrayList of int[]s to int[][]. **arrayListOfDoubleToArray(ArrayList)** - Static method in class util.gen.Num: ArrayList of Double to double[] **arrayListOfFloatToArray(ArrayList)** - Static method in class util.gen.Num: ArrayList of Float to float[] **arrayListOfIntegerToInts(ArrayList)** - Static method in class util.gen.Num: ArrayList of Integer to int[] **arrayListToArray(ArrayList)** - Static method in class bioroot.PatternMatch: Convert an ArrayList of PatternMatch to PatternMatch[] **arrayListToDoubles(ArrayList)** - Static method in class util.gen.Num: ArrayList of Double to double[] **ArrayPrinter** - Class in util.apps: Prints serialized arrays to screen. **ArrayPrinter()** - Constructor for class util.apps.ArrayPrinter: **asciiValue** - Static variable in class edu.utah.seq.parsers.ElandExportLine: **asciScore()** - Static method in class edu.utah.seq.parsers.ElandExportLine: Returns a map of asci text character to it's associated Solexa base quality score. **assembleHeatMapBlocks()** - Method in class edu.utah.seq.data.HeatMapMakerPosNeg: **assembleHeatMapBlocks()** - Method in class trans.main.WindowBlockMakerTwoColor: **assignGenericFeatureTracks()** - Method in class gata.geneGlyphs.Annotation: **assignPValues()** - Method in class expr.CorrelationMaps: Assigns -10Log10(pvalues) for each score > 0 in the CorrelationWindows **aveFoldDiffCombine(int[], int[])** - Static method in class util.gen.Num: Calculates the average fold difference between two int[]s of the same length. **aveFoldDiffIndividual(int[], int[])** - Static method in class util.gen.Num: Calculates the average fold difference between two int[]s of the same length. **aveMedianAbsDiffSubWinControl(Interval)** - Static method in class trans.main.IntervalReportPrinter: Calculate maximum median absolute diff of SubWindow control values **AverageACels** - Class in trans.cel: Averages two serialized float[][]s, like xxx.cela files, from the CelFileConverter app. **AverageACels()** - Constructor for class trans.cel.AverageACels: **averageDoubleArrayList(ArrayList)** - Static method in class selex.UtilSelex: Averages an ArrayList of Double objects. **AverageDoubles** - Class in trans.misc: Slides a 50 index window along an array averaging the contents. **AverageDoubles()** - Constructor for class trans.misc.AverageDoubles: **averageFloatArray(float[])** - Static method in class util.gen.Num: Averages a float[] **averageFloatArrays(float[][])** - Static method in class util.gen.Num: Average float[][] values to double[], averages repeats [values][numRepeats] **averageFloatArraysFlipped(float[][])** - Static method in class util.gen.Num: Average float[][] values to double[], averages repeats [numChips][OligoValues]. **averageFloatArraysFlippedToFloat(float[][])** - Static method in class util.gen.Num: Average float[][] values to double[], averages repeats [numChips][OligoValues]. **averageFloatArraysFlippedToFloats(float[][])** - Static method in class util.gen.Num: Average float[][] values to float[], averages repeats [numChips][OligoValues]. **averageIntArray(int[])** - Static method in class util.gen.Num: Averages a int[] **averageIntegerArrayList(ArrayList)** - Static method in class selex.UtilSelex: Averages an ArrayList of Integer objects. **averageIntIntArray(int[][])** - Static method in class util.gen.Num: Average int[][] values to double[], averages repeats [numRepeats][values] **averageIntIntArray2(int[][])** - Static method in class util.gen.Num: Average int[][] values to double[], averages values [numRepeats][values] **AveragePCels** - Class in trans.cel: Averages serialized float[]s, like xxx.celp files, from the CelProcessor app. **AveragePCels()** - Constructor for class trans.cel.AveragePCels: **averageScores(ExpressedGene[])** - Method in class expr.CorrelationMaps: Average scores \* square root of number of genes. **averageWindowScores(int, int)** - Method in class trans.main.PeakPicker: Averages the smoothed oligo scores, stop is inclusive. **AWS\_ACCESS\_KEY\_PROPERTY\_NAME** - Static variable in class util.amazon.S3CodeSamples: **AWS\_SECRET\_KEY\_PROPERTY\_NAME** - Static variable in class util.amazon.S3CodeSamples: **awsCredentials** - Variable in class util.amazon.S3Manager

---


|  |  |  |
| --- | --- | --- |
| **Overview** \| Package \| Class \| Use \| **Tree** \| **Deprecated** \| **Index** \| **Help** | |  |
| PREV LETTER   **NEXT LETTER** | **FRAMES**    **NO FRAMES**     **All Classes** |  |


A B C D E F G H I J L M N O P Q R S T U V W X Z 

---
